# Current Management and Progress in Radiotherapy for Small Cell Lung Cancer

**DOI:** 10.3389/fonc.2020.01146

**Published:** 2020-07-14

**Authors:** Michael C. Tjong, David Y. Mak, Jeevin Shahi, George J. Li, Hanbo Chen, Alexander V. Louie

**Affiliations:** ^1^Department of Radiation Oncology, Sunnybrook Health Sciences Centre, Toronto, ON, Canada; ^2^Faculty of Medicine, University of Toronto, Toronto, ON, Canada

**Keywords:** small cell lung cancer (SCLC), Radiotherapy—Chemotherapy, review (article), immunotherapy, stereotactic ablative body radiation, radiotherapy—adverse effects

## Abstract

Radiotherapy (RT) and chemotherapy continue to be widely utilized in small cell lung cancer (SCLC) management. In most limited stage (LS)-SCLC cases, the standard initial therapy remains concurrent chemoradiotherapy (CRT), typically with an etoposide and platinum-based regimen. Hyperfractionated twice daily (BID) RT remains the standard of care, though conventional daily (QD) RT is now a viable alternative supported by randomized evidence. In LS-SCLC patients who experienced good response to CRT, prophylactic cranial irradiation (PCI) remains the standard of care. Brain imaging, ideally with MRI, should be performed prior to PCI to screen for clinically apparent brain metastases that may require a higher dose of cranial irradiation. Platinum doublet chemotherapy alone is the historic standard initial therapy in extensive stage (ES)-SCLC. Addition of immunotherapy such as atezolizumab and durvalumab to chemotherapy is now recommended after their benefits were demonstrated in recent trials. In patients with response to chemotherapy, consolidation thoracic RT and PCI could be considered, though with caveats. Emergence of hippocampal avoidance cranial irradiation and SRS in SCLC patients may supplant whole cranial irradiation as future standards of care. Incorporation of novel systemic therapies such as immunotherapies has changed the treatment paradigm and overall outlook of patients with SCLC. This narrative review summarizes the current state, ongoing trials, and future directions of radiotherapy in management of SCLC.

## Introduction

Small cell lung cancer (SCLC) is an aggressive form of lung cancer that accounts for ~15% of all lung cancer diagnoses, with over 30,000 new cases per year in the United States (1–4). Histologically, it is a high-grade neuroendocrine tumor, appearing under the microscope as small round blue malignant cells that stain positive for chromogranin A, synaptophysin and a high Ki-67 index (5–7). It clinically differentiates itself from the more prevalent non-small cell lung cancer (NSCLC) by having a rapid doubling time and high growth rate, with over 70% of patients being diagnosed with metastatic disease at the time of diagnosis (1, 2, 8). Though SCLC is typically responsive to initial therapy, recurrences are common and the prognosis of SCLC patients remains poor with 5-year overall survival rates of under 8% (1, 2, 8).

SCLC is usually categorized as either limited stage (LS) or extensive stage (ES), according to the Veterans' Affairs Lung Study Group (VALSG) classification (9). LS-SCLC is defined as disease that is confined to the ipsilateral hemithorax and regional lymph nodes that can be safely encompassed by a single radiation field, and ES-SCLC consists of the remainder cases that could not be safely treated with radiotherapy initially (10). More recently, the International Association for the Study of Lung Cancer (IASLC) TNM staging system has been shown to further prognosticate SCLC outcomes beyond LS and ES designations, and its use has been recommended for current clinical decision making and clinical trials (11, 12). The use of radiation therapy for SCLC is continuing to evolve due to advances in imaging and radiation delivery techniques. Controversy still exists over the optimal fractionation schedule for concurrent chemoradiotherapy (CRT) in the treatment of LS-SCLC (3, 7, 13). Stereotactic ablative radiotherapy (SABR) is increasingly considered as an alternative to surgery in node-negative LS-SCLC (7, 14). Additionally, prospective data has resulted in enthusiasm to re-evaluate the role of brain MRI surveillance instead of PCI (7, 15). The wider availability of stereotactic radiosurgery (SRS) for brain metastases also raises questions regarding the most appropriate brain-directed radiation strategy (16). This review aims to summarize the current state of radiotherapy in management of LS and ES-SCLC, as well as ongoing clinical trials and future directions.

## Management of Limited Stage-Small Cell Lung Cancer (LS-SCLC)

### Early Stage LS-SCLC

In patients with cT1-2N0 SCLC, surgical resection with lobectomy and mediastinal nodal sampling is recommended as the preferred radical therapy as per National Comprehensive Cancer Network (NCCN) (17, 18). In a National Cancer Data Base (NCDB) propensity-matched review of 2301 cT1-2N0 SCLC patients, surgery and adjuvant chemotherapy were associated with superior overall survival (OS) than concurrent thoracic chemoradiation (CRT) (5-year OS 47.6 vs. 29.8%, *p* < 0.01) (19). Adjuvant chemotherapy without thoracic radiotherapy (TRT) can be given to pN0 and select pN1 patients (20, 21), while patients with pN2 disease should receive thoracic CRT similar to patients with more advanced LS-SCLC at clinical staging (18). For medically inoperable cT1-2 N0 patients, concurrent CRT has been the historical standard. Considering the encouraging results in early stage NSCLC, stereotactic ablative radiotherapy (SABR) is increasingly being utilized for well-staged medically inoperable SCLC patients. In another NCDB study, 2107 histologically confirmed cT1-2N0 SCLC patients did not demonstrate any difference in survival when comparing SABR followed by chemotherapy in those who were eligible, compared to CRT (22). A multi-institutional series of SABR for 74 cT1-2N0 SCLC patients yielded 3-year OS, disease-free survival (DFS), and local control (LC) rates of 34.0, 53.2, and 96.1%, respectively (23). The high rates of LC and relatively high DFS in this series demonstrated the SABR as a standard option for medically inoperable early stage SCLC (23).

### Sequence and Timing of TRT and Chemotherapy

In more advanced LS-SCLC (clinical Stage II-III), concurrent CRT is the current standard of care (18). Concurrent CRT where RT starts with an early cycle (1st or 2nd) of chemotherapy is more effective compared to delayed-start RT or sequential CRT. A non-significant trend toward better survival with concurrent CRT (45 Gy BID at cycle 1 chemotherapy) was shown compared to sequential chemotherapy followed by TRT in a Japan Clinical Oncology Group trial (24). Early TRT yielded better survival compared to delayed TRT (e.g., at cycle 4 of chemotherapy) in 2 meta-analyses (25, 26).

In the meta-analysis by Fried et al., there was a 2-year OS benefit with early TRT, with relative risk (RR): 1.17 (*p* = 0.03), and non-significant trend toward better 3-year OS with RR: 1.13 (*p* = 0.20) when including all seven identified trials. Subset analysis showed the survival benefit was demonstrated in the five trials using platinum-based chemotherapy: 2-year OS RR: 1.30 (*p* = 0.002) and 3-year OS RR: 1.35 (*p* = 0.01), but not in the remaining trials that employed non-platinum chemotherapy (25). In a second meta-analysis by De Ruysscher et al., early TRT did not show OS benefit when including all seven identified trials, but showed significant 5-year OS benefit with odds ratio (OR): 0.64 (*p* = 0.02), and non-significant trend toward better 2-year OS with OR: 0.73 (*p* = 0.07) when excluding 1 trial using non-platinum chemotherapy (26). Shorter period (<30 days) between the start of any treatment until the end of radiotherapy (SER) was shown to predict better 5-year OS, with decrease of 1.83% was shown for each week of SER extension beyond 30 days (27). An updated meta-analysis of individualized patient data from 9 trials further supported early (i.e., within 9 weeks of chemotherapy initiation) and short TRT, but at the cost of increased acute esophagitis (28).

### Optimal Dose and Fractionation

The current standard of care of thoracic CRT dose fractionation was established in the landmark Intergroup 0096 prospective randomized controlled trial (RCT) by Turrisi et al. (29) in 1999, which demonstrated superiority of concurrent hyperfractionated twice daily (BID) TRT (45 Gy/30 fractions BID in 3 weeks) compared to daily (QD) TRT (45 Gy/25 fractions QD in 5 weeks). BID fractionation was associated with an OS benefit (26 vs. 16% at 5 years, *p* = 0.04), but was associated with increased grade 3 acute esophagitis (27 vs. 11%, *p* < 0.001) (29). Following this trial, BID fractionation was not adapted universally. Reasons include the inconvenience of BID treatments and increased toxicity (30, 31). In addition, a common criticism of this trial was that the QD TRT arm employed a lower biologically equivalent dose (BED compared to the BID fractionation (32).

Following this era, two RCTs have compared the Turrisi BID fractionation with higher BED QD regimens (Table 1) (33, 34). The CONVERT trial was reported by Faivre-Finn et al. (34) in 2017, the trial randomized 547 LS-SCLC patients with good performance status to Turrisi BID regimen or QD TRT (66 Gy/33 fractions in 6.5 weeks). The trial was designed with a superiority endpoint for the QD regimen (34). There was no difference in OS between treatment arms, with BID regimen showing a trend toward improved OS (median OS 30 vs. 25 months, *p* = 0.14). Toxicity was similar in both arms (34). The study concluded that BID fractionation should remain the standard of care (32, 34). However, considering the lack of significant survival and toxicity difference between treatment arms, some have argued that the CONVERT QD regimen is a reasonable alternative (35). Considering the lower BED of BID regimen, it is interesting that the CONVERT QD regimen did not improve outcomes. Indeed, as SCLC is both highly proliferative and radiosensitive, malignant repopulation occurs rapidly after each radiation fraction, and therefore may favor shorter time between fractions and a shorter overall RT regimen (32). A CALGB 30610/RTOG 0538 RCT comparing Turrisi regimen against an even higher BED QD regimen (70 Gy/35 fractions in 7 weeks) is ongoing (NCT00632853).

**Table 1:** Selected trials of chemoradiation for LS-SCLC comparing 45 Gy/30 fractions BID regimen with QD TRT regimens.

**Study**	**Completed**	***N***	**TRT in comparison group**	**Chemotherapy (cycles)**	**2-year OS (%)**	**Median OS (months)**	***p*-value**	**Grade 3–4 esophagitis (%)**	***p*-value**
INT-0096 (29)	Yes	471	45 Gy/25 fractions QD	EP (4)	47 (BID) vs. 41 (QD)	23 (BID) vs. 19 (QD)	0.04	32 (BID) vs. 16 (QD)	<0.001
Norwegian Lung Cancer Study Group (33)	Yes	157	42 Gy/15 fractions QD	EP (4)	53 (BID) vs. 42 (QD)	25 (BID) vs. 19 (QD)	0.61	31 (BID) vs. 33 (QD)	0.80
CONVERT (34)	Yes	543	66 Gy/33 fractions QD	EP (4–6)	56 (BID) vs. 51 (QD)	30 (BID) vs. 25 (QD)	0.15	19 (BID) vs. 19 (QD)	0.85
CALGB 30610/RTOG 0538	No	729	70 Gy/35 fractions QD	EP (4)	NA	NA	NA	NA	NA

*BID, twice daily treatments; QD, once daily treatments; EP, etoposide-cisplatin; OS, overall survival*.

Accelerated hypofractionation is a historical option (36) and is still considered a CRT standard option in some parts of the world (37). Recently, a Phase II Scandinavian RCT published by Grønberg et al. in 2015 randomized 157 LS-SCLC patients to either BID (45 Gy/30 fractions BID) or an accelerated hypofractionated QD regimen (42 Gy/15 fractions) (33). The BID fractionation had a numerically higher, though non-significant median OS (25.1 vs. 18.8 months; *p* = 0.61). Furthermore, BID fractionation was associated with higher rates of complete response (CR) compared to QD (33 vs. 13%; *p* = 0.003). There were no differences in severe toxicities. The study's conclusion was that the Turrisi BID fractionation should remain the standard of care (33). Nonetheless proponents of the hypofractionated QD schedule, argue that it is a reasonable option if the BID regimen is logistically difficult, and may be preferred in scenarios wherein a shorter overall treatment course is more viable for the patient.

### Prophylactic Cranial Irradiation in LS-SCLC

Brain metastases (BM) are the most common mode of distant spread in SCLC, with a reported 2-year incidence of approximately 50% among patients not receiving PCI (38, 39). In the Auperin meta-analysis of 7 trials comparing PCI or no PCI among 987 SCLC patients (86% LS-SCLC) with complete response (CR) following initial therapy (57% CRT, 18% chemotherapy alone, 25% chemotherapy ±TRT), the absolute OS benefit of PCI was estimated to be 5.4% at 3 years. The reduction of BM risk was nearly 2-fold [33.3 vs. 58.6%, relative risk (RR): 0.46] (40). The PCI regimens used in the trials were heterogeneous, ranging from 8 Gy/1 fraction to 40 Gy/20 fractions (40). Although this meta-analysis demonstrated the efficacy of PCI in reducing BM and improving OS (41, 42), reasons to withhold PCI include its negative impact on neurocognition and quality of life (QoL) (43, 44). In an RCT of 720 LS-SCLC with CR following CRT, standard-dose PCI (25 Gy/10 fractions) was compared to high-dose PCI (36 Gy/18 fractions), with the standard dose demonstrating improved 2-year OS (42 vs. 37%, *p* = 0.05). The higher PCI dose strategy did not reduce the incidence of BM (23 vs. 29% with standard dose at 2 years, *p* = 0.18) and was also associated with increased neurocognitive toxicity (45).

Given the potential negative effects of PCI on neurocognition, QoL, as well as acute effects such as: nausea, hair loss, and fatigue; there is interest to revisit the role of PCI in LS-SCLC (41). In two series of surgically resected Stage I-III SCLC patients, PCI improved OS for p-Stage II-III, but not p-Stage I patients (39, 46). The lower BM incidence in resected Stage I patients (range 0–15.4%) may explain the purported lack of PCI benefit in this setting (47). These retrospective data suggested that PCI may be omitted in surgically resected p-Stage I SCLC patients, provided that there is brain-directed imaging surveillance (35, 39, 46–48).

The trials in the Auperin meta-analysis were conducted in era prior to the routine use of brain magnetic resonance imaging (MRI) in staging, with CT or clinical neurologic symptoms used to screen for BM prior to PCI (40, 41). Seute et al.'s study demonstrated improved sensitivity of BM detection with MRI (24%, of which 11% were asymptomatic) compared to 10% (all symptomatic) with CT (49). Had patients in the Auperin meta-analysis undergone brain MRI during staging or post-CRT, a proportion may have had BM detected. These patients, therefore, would have received whole brain radiation therapy (WBRT) for undetected, subclinical BM instead of PCI. The use of brain MR surveillance with or without PCI in SCLC patients is the subject of a new Southwest Oncology Group Phase III RCT, MAVERICK (NCT04155034). Based on current data, surgically resected p-Stage I SCLC patients aside, PCI should be offered for all LS-SCLC patients treated with reasonable performance status and no contraindications (e.g., severe cognitive impairment) (47, 48).

## Management of Extensive Stage-Small Cell Lung Cancer (ES-SCLC)

### Consolidative Thoracic Radiation Therapy

The role of TRT is well-established in the management of LS-SCLC, where the early initiation of TRT concurrently with etoposide-carboplatin (EC) or etoposide-cisplatin (EP) has demonstrated improved local tumor control and survival (18, 50, 51). Historically, RT for ES-SCLC was reserved for palliation in the setting of symptomatic locoregional and/or distant disease. The observation that a large proportion of ES patients had recurrent, persistent and/or progressive intrathoracic disease following initial chemotherapy led to a single-institution phase III RCT investigating consolidative TRT in this population (52). In their pivotal RCT, Jeremic et al. randomized 109 patients [with a CR distantly and at least a partial response (PR) in the thorax following 3 cycles of EP] to either further EP alone or consolidative TRT and EP (52). It should be noted that this patient population was carefully selected, with 90% of patients having only 1–2 sites of extrathoracic metastatic disease prior to initial chemotherapy (52, 53). Consolidative TRT (CTRT) was delivered in combination with EC (using an accelerated twice-daily regimen of 54 Gy in 36 fractions) and all patients received prophylactic cranial irradiation (PCI) to a dose of 25 Gy in 10 fractions (52). The investigators found significant improvements in median OS (17 vs. 11 months, *p* = 0.041) and a trend toward improved 5-year local relapse free survival (20 vs. 8.1%; *p* = 0.06) with consolidative TRT (52). Although nearly 1 in four patients (27%) experienced acute grade 3 esophagitis with consolidative TRT, no treatment interruptions were reported, and CTRT was generally well tolerated (52).

Following this RCT, consolidative TRT was not routinely administered following initial chemotherapy, though a few other retrospective and non-randomized prospective studies recapitulated similar findings of a potential benefit as in the Jeremic study (54–57). More recently, the CREST RCT by Slotman et al. randomized 495 ES-SCLC patients with any response to 4–6 cycles of EP to either consolidative TRT (with 30 Gy in 10 fractions) and PCI or PCI alone (58). Although the primary endpoint of 1-year OS was not found to be significantly different between the groups, on secondary analysis, 2-year OS was significantly improved in consolidative TRT patients (13 vs. 3%; *p* = 0.004) (58). Patients receiving consolidative TRT had a near 50% reduction in intrathoracic progression (43.7 vs. 79.8%; *p* < 0.0001) with no significant toxic effects reported (58). In fact, only 4 of 247 patients receiving consolidative TRT experienced grade 3 or greater esophagitis, and the only grade 4 toxicity reported was fatigue in a patient enrolled in the control arm (59), Despite the CREST study not meeting its primary endpoint, the authors concluded that consolidative TRT may improve long-term survival and should be considered for ES-SCLC patients who have had any response to initial chemotherapy (58). This “all-or-none” conclusion drew several criticisms, particularly given the trial's negative primary endpoint, unplanned secondary analysis of 2-year OS, and relatively short median follow-up of 24 months (60). Subgroup analyses of the CREST trial suggest that patients with residual intrathoracic disease (a stratification factor at the time of randomization) benefited the most from consolidative TRT, with a statistically significant difference in OS (HR = 0.81, 95% CI 0.66–0.98, *p* = 0.03) when compared to patients with an intrathoracic CR following chemotherapy (61, 62). In a separate secondary analysis of a subset of CREST patients (89% of whom had intrathoracic residual disease), patients with 2 or fewer metastases had improved OS and progression-free survival (PFS), and the presence of liver and/or bone metastases was a negative prognostic factor for OS (63). These updated analyses suggest that the presence of intrathoracic residual disease, in addition to overall metastatic disease burden, are important factors to consider when identifying ES patients that are most likely to benefit from consolidative TRT (61–63).

Although ES-SCLC generally has a limited prognosis of 8–10 months with chemotherapy alone, Jeremic et al. demonstrated that patients with limited extrathoracic metastatic disease may achieve survival nearing LS-SCLC if PCI and consolidative TRT is delivered (52). This observation, coupled with the finding that disease relapse in patients undergoing multimodality therapy occurs mostly outside of the irradiated brain and thorax, led to the hypothesis that extrathoracic consolidative RT may control limited distant metastases and improve survival (58, 64). To that end, RTOG 0937 was a phase II trial that randomized oligometastatic ES-SCLC patients to either PCI (25 Gy in 10 fractions) or PCI and consolidative RT to the thorax and metastatic sites (30–45 Gy in 10–15 fractions), following a response to initial chemotherapy (64). Unfortunately, the study crossed the futility boundary for the primary endpoint of 1-year OS, and closed after accruing 86 out of a planned 154 patients (64). Recognizing several caveats of a trial that did not complete accrual, as well as imbalances in treatment arms with respect to age, performance status, and disease burden; RTOG 0937 did demonstrate that consolidative RT to residual sites of disease reduced the risk of intrathoracic progression from 83 to 26% (64). Considering that intrathoracic progression in the CREST study was 44% (with 30 Gy in 10 fractions), one interpretation is that higher radiation doses (such as the preferred dose of 45 Gy in 15 fractions used in RTOG 0937) may achieve better local control rates, which may have an effect on survival outcomes. In fact, retrospective series have demonstrated that consolidative TRT doses with a BED with α/β= 10 (BED_10_)> 50 Gy_10_ are associated with improved intrathoracic control and OS (65, 66).

To summarize, future prospective studies should aim to further identify patients routinely benefitting from consolidative TRT, as well as the optimal radiation dose, fractionation, and timing for the safest and most effective treatment. Evidence-based guidelines recommend an individualized approach to clinical decision-making, where consolidative TRT is best suited for patients who respond to initial chemotherapy, present with residual intrathoracic disease, and have minimal extrathoracic disease burden (18, 50). In general, 30 Gy in 10 fractions is considered an acceptable and well-tolerated CTRT dose; however, higher doses may be considered in select patients (18).

### Prophylactic Cranial Irradiation in ES-SCLC

Although more than 50% of patients with SCLC will eventually develop intracranial metastases, the role of PCI in ES-SCLC is often debated, especially in the present era of MRI imaging (53). Although only a minority of the patients in the previously discussed landmark Auperin meta-analysis had ES disease (140 vs. 847 LS patients), subgroup analysis demonstrated a persistent benefit of PCI regardless of the initial extent of disease in patients with a CR to initial chemotherapy with or without TRT (40).

To assess the role of PCI in ES-SCLC, the EORTC conducted a phase III RCT that randomized 286 patients with any response to initial chemotherapy to either PCI (20 Gy in 5–8; 24 Gy in 12; 25 Gy in 10; or 30 Gy in 10–12 fractions) or no additional therapy (67). Pre-treatment brain imaging was not required and was only performed if symptoms of brain metastases were apparent. PCI was found to significantly reduce the incidence of symptomatic brain metastases (15 vs. 40%) and doubled OS (27 vs. 13%) at 1-year (67). A major critique of the EORTC study, however, was that the absence of pre-treatment imaging may have resulted in the treatment of subclinical intracranial metastases with PCI, leading to the modest improvement in median OS observed (6.7 vs. 5.4 months, *p* = 0.003). An additional criticism is the use of several different PCI dose/fractionation regimens, which limits the ability to make conclusions regarding optimal radiation delivery. In terms of tolerability, there was no statistically significant difference between global health status between each arm (*p* = 0.10). Nevertheless, PCI was associated with significantly more fatigue and hair loss, with exploratory analyses demonstrating higher rates of decreased appetite, nausea/vomiting, and leg weakness in those who underwent PCI (*p* < 0.001) (67). Additionally, as many QoL assessments were of low frequency and/or missing due to the overall deterioration of the patients, the EORTC authors commented that the limited number of QoL assessments may have underpowered the ability to detect any potential significant difference in global health status between arms (67).

A subsequent study performed by Japanese investigators addressed many of the concerns raised following the EORTC study (68). In this, phase III RCT, 224 ES-SCLC patients with any response to initial platinum-doublet chemotherapy (and without evidence of brain metastases on MRI) were randomized to PCI (25 Gy in 10 fractions) or MRI surveillance (every 3 months in year 1, and then every 6 months until 24 months) (68). The study was terminated early following an interim analysis of the first 163 patients that revealed futility of the PCI intervention for the primary endpoint of OS. While PCI was found to decrease the incidence of brain metastases (69–48%; *p* < 0.001), there was no difference in median OS (11.6 months with PCI and 13.7 months with observation, *p* = 0.094) (68).

Given the limitations of the EORTC study and the following results of the Japanese trial, a more reserved stance on routine PCI use in ES-SCLC has generally been adopted., Modern surveys of practice patterns indicate that ~50% of radiation oncologists would still offer PCI to ES patients responding to initial chemotherapy (37, 69, 70). Evidence-based guidelines recommend an individualized patient approach, whereby a discussion regarding the potential benefits (e.g., reduced risk for the development of brain metastases) and detriments of PCI (e.g., increased risk of neurocognitive toxicity) should be central to shared clinical decision making (18). In most clinical practices, 25 Gy in 10 fractions appears to be a preferred PCI regimen, with treatment delivered after recovery from initial chemotherapy (18, 50, 51). Higher PCI doses, concurrent chemotherapy, and the treatment of elderly patients and/or those with poor performance status should be avoided given the potential for increased toxicity (18, 37, 45, 70). Hippocampal avoidance and the use of memantine (an NMDA antagonist) have shown promise in reducing neurotoxicity associated with whole-brain RT; though further evidence is required before these techniques become routinely adopted (59, 71, 72). For ES patients undergoing CNS surveillance rather than PCI, it is recommended to perform MRI (preferred) or CT imaging with contrast according to the protocol outlined by Takahashi et al. (68).

## Future Directions of Radiotherapy in SCLC

### Immunotherapy and Radiotherapy in Small Cell Lung Cancer

The advances in cancer immunotherapies have resulted in significant outcome improvements in multiple cancers (73–76). Immunotherapy, predominantly immune-check point inhibitors (ICIs) enhance immune-mediated anticancer activity by blocking immune-attenuating interactions of CTLA-4/B7 or PD-1/PD-L1 receptors between T-lymphocytes and cancer cells (77). Several ongoing studies are being conducted to evaluate the addition of immunotherapy, both concurrently and after CRT for LS-SCLC (Table 2). NRG-LU005 is an active phase II/III trial (NCT03811002) examining the use of atezolizumab with CRT and its effects on PFS and OS. Finally, the ADRIATIC trial—an active phase III, randomized, double-blind, placebo controlled multi-center study (NCT03703297)—that investigates durvalumab and tremelimumab in patients without progression following CRT, with PFS and OS as primary outcomes.

**Table 2:** Selected active clinical trials of immunotherapy and chemoradiation for LS-SCLC.

**ClinicalTrials.gov Trial ID**	**Intervention**	**Primary end point**	**Study status**	**Study design**
NCT03811002	CRT (EP) ± concurrent atezolizumab	PFS, OS	Active, Recruiting	Phase II/III
NCT02402920	CRT (EP) + concurrent pembrolizumab	Pembrolizumab maximum tolerated dose	Active, Recruiting	Phase I
NCT04189094	CRT (EP) ± concurrent sintilimab	PFS at 2 years	Active, not yet recruiting	Phase II
NCT03540420 (ACHILES)	CRT (EP) ± post-treatment atezolizumab	OS at 2 years	Active, Recruiting	Phase II
NCT03585998	CRT (EP) + concurrent and consolidation durvalumab	PFS	Active, Recruiting	Phase II
NCT03703297 (ADRIATIC)	Post-CRT (EP) durvalumab vs. durvalumab + tremelimumab vs. placebo	PFS, OS	Active, Recruiting	Phase III

*CRT, chemoradiotherapy; EP, etoposide-cisplatin; PFS, progression-free survival; OS, overall survival*.

For ES-SCLC, various attempts of combining novel therapies with standard chemotherapy including rilotumumab, ganitumab, and ipilimumab failed to show an OS benefit (78–80), IMpower133 was the first major development of combining ICI and chemotherapy in ES-SCLC, demonstrating significant OS and PFS benefits with the addition of atezolizumab (81). There has been increasing interest to investigate immunotherapy and chemotherapy combinations, such as nivolumab in relapsed ES-SCLC (Checkmate 331), nivolumab ± ipilimumab after chemotherapy (Checkmate 451), ipilimumab alone with chemotherapy (NCT01450761), and pembrolizumab in various regimens (Keynote 028, 159, 604, NCT02359019). Similar to IMpower133, the CASPIAN trial was a three-arm RCT, evaluating the addition of durvalumab with or without tremelimumab to EP chemotherapy. Presently, the arm adding durvalumab has been reported, and demonstrated an improved median OS from 10.3 to 13 months and improved 18-month OS from 25 to 34% (*p*= 0.0047), with no increase in grade 3–4 toxicities (82). Finally, a recently published phase I trial examining pembrolizumab concurrent with the CRT regimen in ES-SCLC demonstrated its safety as a combined regimen, with no grade 4–5 toxicities, and only 6% (*n* = 2) grade 3 adverse effects (83).

A secondary analysis from IMpower133 also demonstrated safety of palliative thoracic radiotherapy among ES-SCLC patients following chemotherapy and immunotherapy. Several ongoing trials are examining the addition of immunotherapy to CRT in ES-SCLC (Table 3). The PICARES study (NCT03971214) is a prospective pilot trial examining consolidation therapy with PD-L1 inhibitors after CRT. Similarly, another phase I trial (NCT02402920) is currently examining the role of concurrent pembrolizumab with RT. Other ongoing studies include examining CRT with nivolumab (NCT03382561) and nivolumab with ipilimumab (NCT03043599), as well as nivolumab and ipilimumab in recurrent ES-SCLC after CRT (NCT03670056). The results of these studies will provide significant insight into the emerging field of combination immuno-chemoradiotherapy, and will help further delineate benefits, dose, timing, toxicities, and indications/contraindications for its use in ES-SCLC.

**Table 3:** Selected active clinical trials of immunotherapy and chemoradiation for ES-SCLC.

**ClinicalTrials.gov Trial ID**	**Intervention**	**Primary end point**	**Study status**	**Study design**
NCT03971214 (PICARES)	Post-CRT (EP) PD-L1 inhibitor	Adverse events, remission rate	Active, not yet recruiting	Phase I
NCT02402920	Post-EP chemotherapy pembrolizumab and BID RT	Safety of pembrolizumab with radiation	Active, Recruiting	Phase I
NCT03043599	Post-EP chemotherapy consolidation ipilimumab and nivolumab with RT	Phase I—Confirmation of ipilimumab and nivolumab dose Phase II—PFS	Active, not recruiting	Phase I/II
NCT03382561	CRT (EP) ± concurrent nivolumab	PFS	Active, not recruiting	Phase II
NCT03670056	Ipilimumab and nivolumab for recurrence after CRT (EP)	Change in ratio of Teff/Treg cells	Active, not recruiting	Phase II

*CRT, chemoradiotherapy; EP, etoposide-cisplatin; BID, twice daily treatments; RT, radiotherapy; PFS, progression-free survival; Teff, effector T-cells; Treg, regulatory T-cells*.

### Hippocampal Avoidance Cranial RT

There has been growing interest in hippocampal-sparing technique during cranial RT to reduce its associated acute side effects, neurocognitive toxicity and QoL detriments (Table 4) (84–89). A phase II trial (90) and subsequent study from Redmond et al. demonstrated that conformal avoidance of the hippocampus during WBRT/PCI was associated with improved memory and QoL, with only 10% of patients developing new BM in the underdosed area, which were amenable to stereotactic radiosurgery (SRS) (59, 90). The recently completed phase III PREMER-TRIAL also demonstrated that compared to conventional PCI, hippocampal avoidance PCI improved free delayed recall at 3 months (21.7 vs. 5.1%), 6 months (32.6 vs. 7.3%), and 12 months (18.5 vs. 3.8%) (91). These encouraging results have led to the development of NRG-CC003, an active, randomized phase II/III trial of WBRT with or without hippocampal avoidance in patients with both LS-SCLC and ES-SCLC (NCT02635009). The trial's primary endpoints are 12-month intracranial relapse and 6-month deterioration of the Hopkins Verbal Learning Test-Revised (HVLT-R) delayed recall.

**Table 4:** Selected active clinical trials of hippocampal avoidance-WBRT/PCI in SCLC.

**ClinicalTrials.gov Trial ID**	**Intervention**	**Primary end point**	**Study status**	**Study design**
NCT02635009	WBRT ± HA in LS and ES-SCLC	Phase II—Intracranial relapse Phase III—Delayed recall deterioration Status	Active, recruiting	Phase II/III
NCT01780675	PCI ± HA in SCLCI-IV	Neurocognitive decline	Active, not recruiting	Phase III
NCT02906384	PCI ± HA in SCLC	Memory preservation and MRI changes	Active, Recruiting	Phase II

*WBRT, whole brain radiation therapy; PCI, prophylactic cranial irradiation; HA, hippocampal avoidance*.

### Stereotactic Radiosurgery in SCLC

The increasing use of SRS in brain metastases has also provided opportunities to examine its efficacy in SCLC (Table 5). Initial studies demonstrated its efficacy in patients who had previously received PCI/WBRT (41, 92–98), with Rava et al. demonstrating that SRS yields excellent LC (81 and 69% at 6 and 12 months, respectively) for small lesions <2 cm (99). Some studies supported the viability of omitting of PCI/WBRT in favor of active MRI surveillance and SRS as first-line therapy for emerging brain metastases (41, 97, 98, 100, 101). Ozawa et al. demonstrated that MRI surveillance and SRS for BM had an equivalent OS to initial PCI for LS-SCLC (102), while Chang et al. demonstrated that SRS alone without PCI/WBRT is associated with better neurocognition, learning and memory function (84). Nonetheless, as of 2020, the NCCN guidelines do not suggest the use of SRS alone given the high rate of brain metastases in SCLC (18). Rather, SRS is preferred (if feasible) in patients who develop BM after PCI, particularly if there is a prolonged time between PCI and BM occurrence and if extracranial disease is controlled (18). The results of ongoing trials will further inform the role of SRS in SCLC patients. In particular, ENCEPHALON (NCT03297788) is an ongoing phase II trial examining WBRT vs. SRS for SCLC with 1–10 BM. Similarly, NCT03391362 is single arm, phase II trial examining SRS in SCLC pts with 1–6 BM. Investigations of SRS with other therapies are also ongoing, such as the use of SRS and nivolumab (NCT02978404) and SRS with the medical device NovoTTF-200A (NCT03488472). NovoTTF-200A is a battery-operated, portable device that produces changing electrical fields (known as Tumor Treatment Fields) through ceramic disks placed on the head to stop the growth of brain tumor cells, and potentially sensitize tumor cells to immunotherapies. An active ongoing trial is currently examining its use, feasibility, and compliance in ES-SCLC for prevention of BM (NCT03607682).

**Table 5:** Selected active clinical trials of brain SRS in SCLC.

**ClinicalTrials.gov Trial ID**	**Intervention**	**Primary end point**	**Study status**	**Study design**
NCT03297788 (ENCEPHALON)	WBRT vs. SRS for 1-10 SCLC-BM	Neurocognition	Active, recruiting	Phase II
NCT02978404	SRS + nivolumab for SCLC-BM	Intracranial PFS	Active, recruiting	Phase II
NCT03391362	SRS for 1–6 SCLC-BM	Death due to progressive neurologic disease	Active, recruiting	Phase II

*SRS, stereotactic radiosurgery; BM, brain metastases; PFS, progression-free survival*.

SCLC continues to be associated with poor prognosis. However, there continues to be promising progress in its multidisciplinary management involving radiotherapy, systemic therapies, medical imaging, and surgery. While randomized data supports the addition of immunotherapy to standard chemotherapy in extensive stage disease, its role in limited stage disease has not yet been established. In addition, novel applications of radiation technologies such as SABR and hippocampal avoidance cranial irradiation hold promise for the radical, palliative, and preventative management of this disease.

## Author Contributions

MT: designing and planning of the review, conducting literature review, synthesis of literature findings, writing, and editing manuscript. AL: supervising the project, designing and planning of the review, conducting literature review, synthesis of literature findings, writing, and editing manuscript. HC, GL, JS, and DM: conducting literature review, synthesis of literature findings, writing, and editing manuscript. All authors contributed to the article and approved the submitted version.

## Conflict of Interest

AL has received honoraria from RefleXion, Varian Medical Systems Inc. and AstraZeneca. The remaining authors declare that the research was conducted in the absence of any commercial or financial relationships that could be construed as a potential conflict of interest.
